# Intracranial VCAM1 at time of mechanical thrombectomy predicts ischemic stroke severity

**DOI:** 10.1186/s12974-021-02157-4

**Published:** 2021-05-11

**Authors:** Benton Maglinger, Madison Sands, Jacqueline A. Frank, Christopher J. McLouth, Amanda L. Trout, Jill M. Roberts, Stephen Grupke, Jadwiga Turchan-Cholewo, Ann M. Stowe, Justin F. Fraser, Keith R. Pennypacker

**Affiliations:** 1 Department of Neurology, University of Kentucky, Lexington, KY USA; 2 Center for Advanced Translational Stroke Science, University of Kentucky, Lexington, KY 40536 USA; 3 Department of Behavioral Science, University of Kentucky, Lexington, KY USA; 4 Department of Neuroscience, University of Kentucky, Lexington, KY USA; 5 Department of Neurosurgery, University of Kentucky, Lexington, KY USA; 6 Department of Neurosurgery and Neuroendovascular Surgery, Covenant Medical Center, Lubbock, TX USA; 7 Department of Radiology, University of Kentucky, Lexington, KY USA

**Keywords:** Vascular cell adhesion molecule 1, Ischemic stroke, Infarct volume, Mechanical thrombectomy, Biomarkers, Proteomics

## Abstract

**Background:**

Emergent large vessel occlusion (ELVO) strokes are devastating ischemic vascular events for which novel treatment options are needed. Using vascular cell adhesion molecule 1 (VCAM1) as a prototype, the objective of this study was to identify proteomic biomarkers and network signaling functions that are potential therapeutic targets for adjuvant treatment for mechanical thrombectomy.

**Methods:**

The blood and clot thrombectomy and collaboration (BACTRAC) study is a continually enrolling tissue bank and registry from stroke patients undergoing mechanical thrombectomy. Plasma proteins from intracranial (distal to clot) and systemic arterial blood (carotid) were analyzed by Olink Proteomics for *N*=42 subjects. Statistical analysis of plasma proteomics used independent sample *t* tests, correlations, linear regression, and robust regression models to determine network signaling and predictors of clinical outcomes. Data and network analyses were performed using IBM SPSS Statistics, SAS v 9.4, and STRING V11.

**Results:**

Increased systemic (*p*<0.001) and intracranial (*p*=0.013) levels of VCAM1 were associated with the presence of hypertension. Intracranial VCAM1 was positively correlated to both infarct volume (*p*=0.032; *r*=0.34) and edema volume (*p*=0.026; *r*=0.35). The %∆ in NIHSS from admittance to discharge was found to be significantly correlated to both systemic (*p*=0.013; *r* = −0.409) and intracranial (*p*=0.011; *r* = −0.421) VCAM1 levels indicating elevated levels of systemic and intracranial VCAM1 are associated with reduced improvement of stroke severity based on NIHSS from admittance to discharge. STRING-generated analyses identified biologic functional descriptions as well as function-associated proteins from the predictive models of infarct and edema volume.

**Conclusions:**

The current study provides novel data on systemic and intracranial VCAM1 in relation to stroke comorbidities, stroke severity, functional outcomes, and the role VCAM1 plays in complex protein-protein signaling pathways. These data will allow future studies to develop predictive biomarkers and proteomic targets for drug development to improve our ability to treat a devastating pathology.

**Supplementary Information:**

The online version contains supplementary material available at 10.1186/s12974-021-02157-4.

## Background

Vascular cell adhesion molecule 1 (VCAM1) mediates leukocyte-endothelial cell adhesion and has been associated with pathologies such as ischemic stroke [1]. It is known that cerebrovascular ischemia upregulates leukocyte-endothelial adhesion molecules [2–6]. Upregulation of these proteins permits inflammatory cells to adhere to and migrate through the vascular wall. Ischemic events exacerbate tissue injury in part by inducing endothelial damage allowing leukocytes to extravasate into brain tissue [7]. Polymorphonuclear leukocytes (PMNs) aggravate the injured tissue both at the site of ischemia as well as the peripheral penumbra [8, 9]. This extravasation process contributes to consequential inflammation and vasogenic edema leading to the worsening of the impact of ischemic insult [10, 11].

The University of Kentucky Center for Advanced Translational Stroke Science utilizes mechanical thrombectomy to better understand emergent large vessel occlusion (ELVO) stroke. Following the blood and clot thrombectomy registry and collaboration (BACTRAC) protocol (clinicaltrials.gov NCT03153683), human tissue samples are banked and subsequently used for experimentation [12]. This protocol allows for isolation of intracranial (i.e., distal to thrombus) arterial blood and systemic (i.e., carotid) arterial blood from thrombectomy procedures. To date, we have studied arterial blood gasses (ABGs), electrolyte chemistry, genomics, neuroinflammation, and proteomics [13–15].

The objective of this study was to assess VCAM1 protein concentrations as a potential biomarker of injury. We selected VCAM1 as our prototype protein for several reasons. First, VCAM1 is an example of an endpoint protein downstream from critical proinflammatory signals such as TNFα and IL-6. Secondly, the function of VCAM1 is particularly relevant to neuroinflammation in stroke as it acts to permit extravasation of leukocytes through the vascular wall into brain tissue. Lastly, VCAM1 is well-reported in the literature in the context of ischemia, vascular comorbidities, and molecular signaling pathways which allows us to compare our data to published benchmarks. The first goal was to assess how intracranial and systemic proteomic expression levels of VCAM1 differed according to comorbid conditions such as hypertension (HTN), smoking, and hyperlipidemia. Second, we investigated the relationships of systemic and intracranial VCAM1 with metrics of stroke severity and functional recovery including infarct volume, infarct edema, infarct time, and National Institute of Health Stroke Scale/Score (NIHSS). Lastly, we analyzed VCAM1 data in context to related cardiometabolic and inflammatory proteins to elucidate relevant proteomic signaling networks involved in ischemic stroke. One specific protein relationship studied was interleukin 6 (IL-6) as it has been shown to be related to stroke risk factors, infarct volume, and clinical outcomes [16, 17]. These data will elucidate predictive biomarkers and potential therapeutic targets for the prognostics and treatment of ELVO patients.

## Methods

### Sample acquisition and analysis

The BACTRAC program is a continually enrolling human tissue bank and registry (clinicaltrials.gov NCT03153683) of patients undergoing mechanical thrombectomy (MT) for emergent large vessel occlusion (ELVO) stroke. The study is approved through the Institutional Review Board (IRB) at the University of Kentucky; written consent is obtained from the subjects or their legally authorized representative. All non-pregnant adult (18 or older) thrombectomy candidates were considered for inclusion. The main exclusions occurred due to patients either being pregnant, imprisoned, or unable to consent within a 72-h window outlined by the IRB. While the tissue bank is continuously enrolling, samples included in the present study were obtained between June 21, 2017, and August 2, 2020. Methods in which tissue samples were obtained during thrombectomy have been previously published [12]. Intracranial and systemic plasma samples from 42 subjects underwent cardiometabolic and inflammatory proteomic analysis at Olink Proteomics (Olink Proteomics, Boston, MA). We chose Olink specifically for their well-established methods regarding proteomic analyses on small-volume samples. This is of particular interest to us as our intracranial samples are small volume in nature. Olink utilizes a unique proximity extension assay (PEA) technology and rather than reporting protein expression in a concentration value, they report a Normalized Protein eXpression (NPX) value that is a unit in log2 scale which allows for individual protein analysis across a sample set. Methods outlining tissue processing and protein isolation has also been previously published [15]. Demographic data including age, sex, BMI, hypertension, diabetes, hyperlipidemia, previous stroke, previous myocardial infarction, smoking status, NIHSS on admission, NIHSS on discharge, thrombolysis in cerebral infarction (TICI) score, computed tomography angiogram (CTA) collateral score, infarct time, infarct volume, and edema volume are reported for each subject. The % change in NIHSS was calculated as ((Admittance NIHSS − Discharge NIHSS)/Admittance NIHSS).

### Statistical analysis

Two patients had significantly smaller infarct volumes (448 mm^3^ and 560 mm^3^ compared to mean of 56,919 mm^3^) and edema volumes (0 mm^3^ and 220 mm^3^ compared to mean of 60,529 mm^3^) than the rest of the patient population. These two subjects were found to be statistical outliers with high leverage values and were removed from *t* tests, correlations, and linear regression analyses, and are not reported in the demographics section. For all analyses conducted in this study except the robust regression, *N*=40. Since robust regression takes into account the outliers and their influence on statistical outputs, these analyses were conducted with all 42 subjects (see below for details). Independent sample *t* tests were used to investigate relationships between VCAM1 expression and categorical variables. Correlation coefficients were used to assess the relationship between VCAM1 expression and continuous outcomes. Due to a non-normal distribution, both infarct and edema volume were log transformed to achieve normality. NIHSS data were analyzed in two separate ways. First, *t* tests were conducted for NIHSS on admittance relative to the median NIHSS admittance score, which was 18. NIHSS on discharge was treated similarly relative to a median of 9. In addition to these analyses, NIHSS was also broken down into 4 statistical quartiles each containing 10 subjects. From lowest to highest, these quartiles were labeled as “mild stroke,” “mild to moderate stroke,” “moderate to severe stroke,” and “severe stroke.”

A multiple linear regression-based approach was used to determine the network of proteins predictive of infarct and edema volume. Regression diagnostics determined the presence of several outliers for infarct and edema volume, as well as high leverage values for some of the proteins. To account for this, robust regression with MM estimation was used. In this method, observations with high outliers and leverage values are given less weight in the analysis allowing for data analysis on all 42 subjects. In order to determine the set of proteins accounting for the most variability in infarct and edema volume, a forward selection approach was used. In the first step, VCAM1 was entered as a predictor. In the subsequent steps, the protein with the next strongest association with infarct or edema volume was entered, until 10 additional proteins were selected. A final model using these 11 proteins was then assessed. Some rules of thumb suggest that the number of subjects per variable in linear regression models should be between five and twenty to avoid overfitting the model. However, a recent simulation study found that low bias and accurately estimated standard errors can be achieved with as little as two subjects per variable [18]. For the current study, a subject per variable ratio of over 4 was determined to mitigate the risk of overfitting. Network analysis was performed on STRING V11 (https://string-db.org/) using protein-protein interaction analysis data. All statistical analyses were performed using SAS v 9.4 (SAS Institute Inc., Cary, NC) or IBM SPSS Statistics.

## Results

### Patient characteristics

Subject demographic data are shown in Table 1. There were 40 adult (>18 years of age) subjects included in the study with a median age of 67 (25-96), of which 24 (60%) were female. Sixteen subjects had a normal body mass index (BMI), 17 were overweight, and 7 were obese. Of note, 10 (26%) were current smokers, and 6 (16%) were previous smokers. According to the National Institutes of Health Stroke Score (NIHSS), on admission, 1 (3%) of the patients had a minor stroke (NIHSS 1-4), 15 (38%) were considered to have a moderate stroke (NIHSS 5-15), 11 (28%) were considered to have a moderate/severe stroke (NIHSS 16-20), and 12 (31%) were considered to have a severe stroke (NIHSS > 21). On discharge, 13 (34%) were considered to have a minor stroke, 17 (45%) were considered to have a moderate stroke, 5 (13%) were considered to have a moderate/severe stroke (NIHSS 16-20), and 3 (8%) were considered to have a severe stroke (NIHSS > 21). The mean last known normal (LKN) to thrombectomy completion time was 639 + 367 min and the mean infarct volume was 56,739 + 74,880 mm^3^.

**Table 1:** Demographics and characteristics for thrombectomy subjects (*N*=40)

	Value (%)
**Age (median; range)**	67 (25-96)
**Sex**	
Female	24 (60)
Male	16 (40)
**BMI**	
<24.9	16 (40)
25-29.9	17 (42.5)
30-39.9	7 (17.5)
>40	0 (0)
**Comorbidities**	
Hypertension	28 (70)
Diabetes mellitus II	9 (22.5)
Hyperlipidemia	11 (27.5)
Previous stroke	8 (20)
Smoking status**	
Never	22 (58)
Currently	10 (26)
Previously (> 6 months)	6 (16)
NIHSS on admission*	
Minor stroke (1–4)	1 (3)
Moderate stroke (5–15)	15 (38)
Moderate/severe (16–20)	11 (28)
Severe stroke (≥ 21)	12 (31)
NIHSS at discharge**	
Minor stroke (1–4)	13 (34)
Moderate stroke (5–15)	17 (45)
Moderate/severe (16–20)	5 (13)
Severe stroke (≥ 21)	3 (8)
**TICI Score**	
2A = < 50% perfusion	1 (2.5)
2B = > 50% perfusion	20 (50)
3 = full perfusion	19 (47.5)
**LKN to thrombectomy**	
Completion time (minutes)**	639.87 + 367
**Infarct volume (mm**^**3**^**)**	56,739 + 74,880
CTA collateral score**	
0	8 (21)
1	24 (63)
2	5 (13)
3	1 (3)

Values are median with range, mean ± SD, or (%)

*N*=40 patients

*1 patient’s data missing (*n*=39)

**2 patient’s data missing (*n*=38)

### VCAM1 and comorbidities

In our cohort of ELVO ischemic stroke patients, proteomic expression of systemic and intracranial VCAM1 was studied relative to patient sex and comorbid conditions such as hypertension, dyslipidemia, and smoking status. Increased levels of systemic VCAM1 were significantly related to the presence of HTN (mean without HTN = 5.17, SD = 0.28, mean with HTN = 5.80, SD = 0.50, and *p* value = <0.001). Similarly, increased levels of intracranial VCAM1 were significantly related to the presence of HTN (mean without HTN = 5.06, SD = 0.30, mean with HTN = 5.54, SD = 0.70, and *p* value = 0.005). We found no significant relationship with hyperlipidemia and systemic (*p*=0.111) or intracranial (*p*=0.091) VCAM1. There were also no significant relationships between systemic or intracranial VCAM1 expression levels and whether or not the patient was a smoker (*p*=0.962 and *p*=0.841, respectively). Finally, there were also no significant relationships when looking at sex versus systemic VCAM1 (*p*=0.632) or intracranial VCAM1 (*p*=0.754).

### VCAM1 versus stroke severity and functional recovery after thrombectomy

**Fig. 1:**
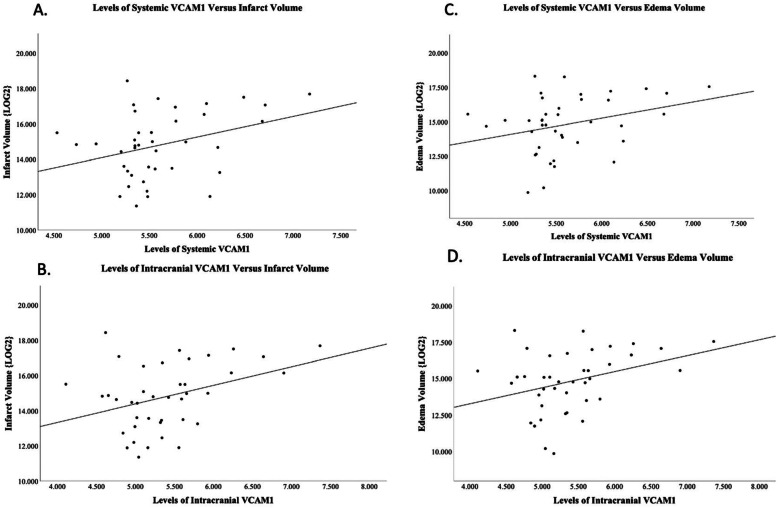
Spearman correlations for VCAM1 and infarct volume and edema volume. **a** SystemicVCAM1 levels vs. infarct volume log2 transformed (*p*=0.07; *r*=0.28). **b** Intracranial VCAM1 levels vs. infarct volume log2 transformed (*p*=0.032; *r*=0.34). **c** Systemic VCAM1 levels vs. edema volume log2 transformed (*p*=0.09; *r*=0.27). **d** Intracranial VCAM1 levels vs. edema volume log2 transformed (*p*=0.026; *r*=0.35)

Correlations between VCAM1 and infarct volume and edema volume can be found in Fig. 1a-d. Intracranial VCAM1 was positively correlated to both infarct volume (*p*=0.032; *r*=0.34) and edema volume (*p*=0.026; *r*=0.35). Systemic VCAM1 was found to have near-significant positive correlations with both infarct volume (*p*=0.07; *r*=0.28) and edema volume (*p*=0.09; *r*=0.27).

The %∆ in NIHSS from admittance to discharge was found to be significantly correlated to both systemic (*p*=0.013; *r* = −0.409) and intracranial (*p*=0.011; *r* = −0.421) VCAM1 levels indicating elevated levels of systemic and intracranial VCAM1 are associated with reduced improvement of stroke severity based on NIHSS from admittance to discharge (data not graphed). Intracranial and systemic VCAM1 levels were also assessed based on median NIHSS on admittance and discharge. The median NIHSS for admission was 18 and median NIHSS for discharge was 9. On admission, relative to a median NIHSS of 18, those with NIHSS > 18 had higher systemic VCAM1 levels as compared to the systemic VCAM1 levels in subjects with NIHSS admittance score <18 (mean for NIHSS >18 = 5.8, SD = 0.68; mean for NIHSS <18 = 5.4, SD = 0.27; *p* value = 0.059). Those with NIHSS >18 had higher intracranial VCAM1 levels as compared to the intracranial VCAM1 levels in subjects with NIHSS admittance score <18 (mean NIHSS for >18 = 5.6, SD = 0.76; mean NIHSS <18 = 5.2, SD = 0.44; *p* value = 0.058). Although near-significant, admission data did not reach *p* value <0.05. On discharge, however, relative to a median NIHSS of 9, those with NIHSS >9 had significantly higher systemic VCAM1 levels as compared to the systemic VCAM1 levels in subjects with NIHSS discharge score <9 (mean for NIHSS >9 = 5.8, SD = 0.47; mean for NIHSS <9 = 5.4, SD = 0.53; *p* value = 0.009). No significant relationship was identified for intracranial VCAM1 levels and discharge NIHSS. Lastly, the relationship between VCAM1 and NIHSS was also assessed by breaking our cohort into statistical quartiles. The 10 subjects with the lowest NIHSS scores (mild stroke category) were compared to the 10 subjects with the highest NIHSS scores (severe stroke category). Systemic VCAM1 levels were found to be significantly higher in the severe stroke quartile as compared to the mild stroke quartile (mean for mild = 5.4, SD = 0.22; mean for severe = 5.7, SD = 0.43; *p* value = 0.024). We found no significant relationship with intracranial VCAM1 levels (mean for mild = 5.2, SD = 0.45; mean for severe = 5.5, SD = 0.43; *p* value = 0.181). Finally, in our cohort of subjects, there were no significant relationships between either systemic or intracranial VCAM1 and infarct time (time from last known normal) with *p*=0.145 and *p*=0.404, respectively.

### VCAM1 and signaling proteins

All protein names and functions are directly extracted from Olink and STRING and are provided in Supplemental Table 1. Systemic VCAM1 was significantly correlated to both systemic interleukin 6 (IL-6) as well as intracranial IL-6 (*p*=0.006 and *p*=0.001, respectively). Intracranial VCAM1 was significantly correlated to intracranial IL-6 but not systemic IL-6 (*p*=0.003 and *p*=0.098, respectively).

Robust regressions were used to determine systemic and intracranial proteins most predictive of infarct volume and edema volume in the context of VCAM1. The top 10 most predictive proteins identified by each model are shown in Table 2. The *R*^2^ values for the four predictive models were systemic proteins and infarct volume *R*^2^= 0.7551, intracranial proteins and infarct volume *R*^2^=0.6665, systemic proteins and edema volume R^2^=0.6396, and intracranial proteins and edema volume *R*^2^=0.6564.

**Table 2:** Proteins with strongest predictive value for infarct volume or edema volume based on location of blood sample. For directionality, if the relationship is positive, higher expression levels of that protein is associated with higher infarct/edema volume. If the relationship is negative, higher levels of that protein are associated with lower infarct/edema volume. Included in the table are *R*^2^ values for each predictive model

Protein	*p* value	Directionality
Systemic blood and infarct volume *R*^2^=0.7551		
APOM	<0.0001	(−)
MMP10	0.0006	(+)
DPP4	<0.0001	(−)
CST3	<0.0001	(−)
IL10RB	<0.0001	(+)
AOC3	0.0191	(+)
CXCL9	<0.0001	(+)
VASN	0.0009	(+)
IL20RA	0.0038	(+)
IL18	0.0182	(+)
Intracranial blood and infarct volume *R*^2^=0.6665		
SLAMF1	<0.0001	(+)
CCL18	<0.0001	(+)
NCAM1	<0.0001	(+)
LILRB2	0.0359	(−)
CD59	<0.0001	(+)
IGFBP6	<0.0001	(−)
ANG	<0.0001	(−)
MEGF9	0.0087	(−)
MCP1	0.0001	(−)
ITGAM	0.0053	(−)
Systemic blood and edema volume *R*^2^=0.6396		
APOM	0.0014	(−)
MMP10	0.0014	(+)
NT3	<0.0001	(−)
PDL1	<0.0001	(−)
SLAMF1	<0.0001	(+)
CCL5	<0.0001	(+)
ANG	<0.0001	(−)
CHL1	<0.0001	(−)
ITGAM	<0.0001	(−)
CCL25	<0.0001	(+)
Intracranial blood and edema volume *R*^2^=0.6564		
CCL18	<0.0001	(+)
SLAMF1	<0.0001	(+)
FAP	<0.0001	(−)
PDL1	<0.0001	(−)
TSLP	0.1851	(+)
F7	<0.0001	(−)
MBL2	0.0001	(−)
CSF1	0.0045	(−)
CCL11	0.0012	(−)
IL15RA	0.0261	(+)

**Fig. 2:**
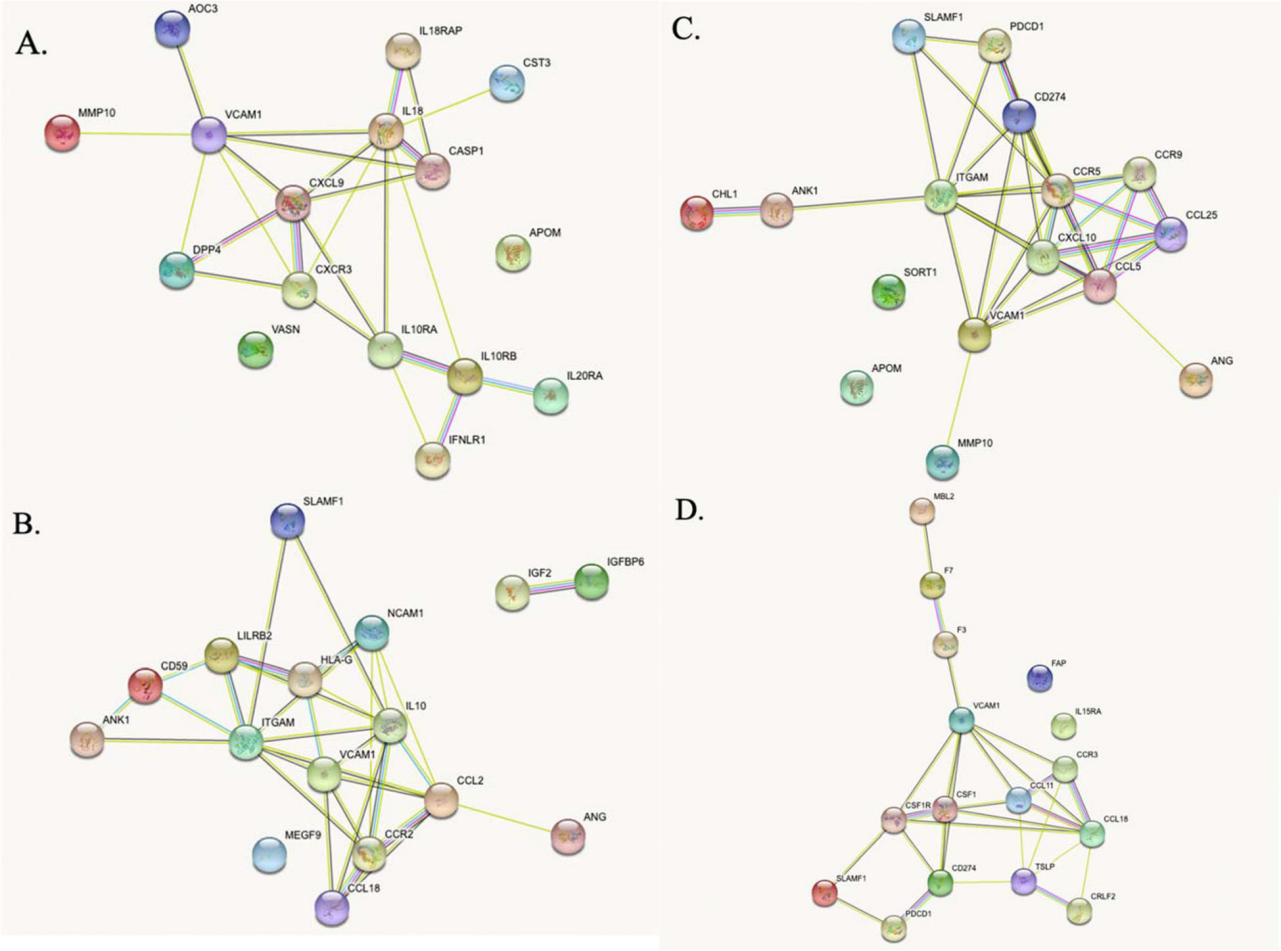
STRING protein-protein network analyses for (**a**) systemic VCAM1 and infarct volume, (**b**) intracranial VCAM1 and infarct volume, (**c**) systemic VCAM1 and infarct edema volume, and (**d**) intracranial VCAM1 and infarct edema volume

The 10 proteins identified by each predictive model were used as the input for STRING analysis. STRING analyses report physical and functional associations between proteins by utilizing genome-wide proteomic connectivity integrations. STRING network webs based on systemic and intracranial predictors of infarct volume and edema volume are shown in Fig. 2. STRING-generated functional descriptions and associated proteins are shown in Table 3. This table illustrates the top 10 network functions for each predictive model along with the corresponding proteins for each specific function. Ranking of functions into top 10 was done based on smallest false discovery rates (FDRs).

**Table 3:** Network functions and associated proteins

Term ID	Biological process	Strength	False discovery rate	Matching proteins in network
**A. Systemic VCAM1 and infarct volume network**				
GO:0019221	Cytokine-mediated signaling pathway	1.25	1.87E−07	IL10RA, IL18RAP, IL18, IL10RB, IL20RA, IFNLR1, CXCL9, CXCR3, CASP1
GO:0070887	Cellular response to chemical stimulus	0.8	1.89E−07	IL10RA, IL18RAP, IL18, IL10RB, VASN, AOC3, IL20RA, IFNLR1, CXCL9, CXCR3, APOM, CST3, CASP1
GO:0006952	Defense response	1.02	6.16E−07	IL18RAP, IL18, IL10RB, AOC3, IFNLR1, DPP4, CXCL9, CXCR3, CST3, CASP1
GO:0010033	Response to organic substance	0.71	4.92E−05	IL10RA, IL18RAP, IL18, IL10RB, IL20RA, IFNLR1, CXCL9, CXCR3, APOM, CST3, CASP1
GO:0006954	Inflammatory response	1.21	9.58E−05	IL18RAP, IL18, IL10RB, AOC3, CXCL9, CXCR3
GO:0006950	Response to stress	0.64	0.00018	IL18RAP, IL18, IL10RB, VASN, AOC3, IFNLR1, DPP4, CXCL9, CXCR3, CST3, CASP1
GO:0001816	Cytokine production	1.58	0.00026	IL18RAP, IL18, CXCR3, CASP1
GO:0010716	Negative regulation of extracellular matrix disassembly	2.81	0.00056	DPP4, CST3
GO:0035744	T-helper 1 cell cytokine production	2.81	0.00056	IL18RAP, IL18
GO:0002376	Immune system process	0.69	0.00076	IL18RAP, IL18, IL10RB, IFNLR1, DPP4, CXCL9, CXCR3, CST3, CASP1
**B. Intracranial VCAM1 and infarct volume networks**				
GO:0002696	Positive regulation of leukocyte activation	1.52	4.66E−08	CCL2, CCR2, VCAM1, LILRB2, IGF2, IL10, HLA-G, ITGAM
GO:0006955	Immune response	0.97	4.66E−08	CCL2, CCR2, VCAM1, SLAMF1, ANG, LILRB2, CD59, IL10, HLA-G, ITGAM, CCL18, NCAM1
GO:0006952	Defense response	1.04	6.64E−08	CCL2, CCR2, VCAM1, SLAMF1, ANG, LILRB2, IL10, HLA-G, ITGAM, CCL18, NCAM1
GO:0022409	Positive regulation of cell-cell adhesion	1.56	1.14E−07	CCL2, CCR2, VCAM1, LILRB2, IGF2, IL10, HLA-G
GO:1903037	Regulation of leukocyte cell-cell adhesion	1.49	2.67E−07	CCL2, CCR2, VCAM1, LILRB2, IGF2, IL10, HLA-G
GO:0042129	Regulation of T cell proliferation	1.69	2.81E−07	CCR2, VCAM1, LILRB2, IGF2, IL10, HLA-G
GO:0050863	Regulation of T cell activation	1.45	3.15E−07	CCL2, CCR2, VCAM1, LILRB2, IGF2, IL10, HLA-G
GO:0051707	Response to other organism	1.02	4.04E−07	CCL2, VCAM1, SLAMF1, ANG, LILRB2, IL10, HLA-G, ITGAM, CCL18, NCAM1
GO:0098542	Defense response to other organism	1.11	4.87E−07	CCL2, VCAM1, SLAMF1, ANG, IL10, HLA-G, ITGAM, CCL18, NCAM1
GO:0050870	Positive regulation of T cell activation	1.58	6.23E−07	CCL2, CCR2, VCAM1, LILRB2, IGF2, HLA-G
**C. Systemic VCAM1 and edema volume networks**				
GO:0006955	Immune response	0.94	2.65E−06	CCR5, VCAM1, CXCL10, SLAMF1, PDCD1, ANG, CCR9, CD274, CCL25, ITGAM, CCL5
GO:0070098	Chemokine-mediated signaling pathway	1.91	2.65E−06	CCR5, CXCL10, CCR9, CCL25, CCL5
GO:0006935	Chemotaxis	1.24	1.28E−05	CHL1, CCR5, VCAM1, CXCL10, CCR9, CCL25, CCL5
GO:0006952	Defense response	0.95	1.28E−05	CCR5, VCAM1, CXCL10, SLAMF1, ANG, CCR9, CCL25, ITGAM, CCL5
GO:0016477	Cell migration	1.08	1.28E−05	CHL1, CCR5, VCAM1, CXCL10, ANG, CCL25, ITGAM, CCL5
GO:0040011	Locomotion	0.98	1.28E−05	CHL1, CCR5, VCAM1, CXCL10, ANG, CCR9, CCL25, ITGAM, CCL5
GO:0050900	Leukocyte migration	1.39	1.28E−05	CCR5, VCAM1, CXCL10, CCL25, ITGAM, CCL5
GO:0060326	Cell chemotaxis	1.52	2.29E−05	CCR5, VCAM1, CXCL10, CCL25, CCL5
GO:0070234	Positive regulation of T cell apoptotic process	2.39	2.50E−05	PDCD1, CD274, CCL5
GO:0019221	Cytokine-mediated signaling pathway	1.12	2.77E−05	CCR5, VCAM1, CXCL10, CCR9, CCL25, ITGAM, CCL5
**D. Intracranial VCAM1 and edema volume networks**				
GO:0019221	Cytokine-mediated signaling pathway	1.27	1.07E−08	CSF1R, VCAM1, CCL11, CSF1, F3, TSLP, IL15RA, CRLF2, CCR3, CCL18
GO:0034097	Response to cytokine	1.11	1.38E−08	CSF1R, VCAM1, CCL11, CSF1, F3, TSLP, CD274, IL15RA, CRLF2, CCR3, CCL18
GO:0008284	Positive regulation of cell population proliferation	1.14	6.15E−08	CSF1R, VCAM1, CCL11, SLAMF1, CSF1, F3, TSLP, CD274, CRLF2, CCR3
GO:0006954	Inflammatory response	1.31	2.65E−07	CSF1R, VCAM1, CCL11, CSF1, F3, MBL2, CCR3, CCL18
GO:0042127	Regulation of cell population proliferation	0.93	4.53E−07	FAP, CSF1R, VCAM1, CCL11, SLAMF1, CSF1, F3, TSLP, CD274, CRLF2, CCR3
GO:0006952	Defense response	1	7.01E−07	CSF1R, VCAM1, CCL11, SLAMF1, CSF1, F3, TSLP, MBL2, CCR3, CCL18
GO:0048522	Positive regulation of cellular process	0.57	1.25E−06	FAP, CSF1R, VCAM1, CCL11, SLAMF1, CSF1, F3, PDCD1, TSLP, MBL2, F7, CD274, CRLF2, CCR3, CCL18
GO:1902533	Positive regulation of intracellular signal transduction	1.06	1.33E−06	CSF1R, CCL11, SLAMF1, CSF1, F3, TSLP, F7, CRLF2, CCL18
GO:0048584	Positive regulation of response to stimulus	0.82	3.90E−06	CSF1R, CCL11, SLAMF1, CSF1, F3, TSLP, MBL2, F7, CD274, CRLF2, CCL18
GO:0006955	Immune response	0.89	4.19E−06	CSF1R, VCAM1, CCL11, SLAMF1, CSF1, PDCD1, TSLP, MBL2, CD274, CCL18

STRING Term ID provided in the first column. Top 10 biological functions for each network based on smallest false discovery rate (FDR) listed in second column. Analysis strength and FDR listed in columns 3 and 4, respectively. Column 5 lists function-related proteins for each biologic process. Table is broken down by network: (A) Systemic VCAM1 and infarct volume network, (B) Intracranial VCAM1 and infarct volume network, (C) Systemic VCAM1 and infarct edema volume network, and (D) Intracranial VCAM1 and infarct edema volume network

## Discussion

VCAM1 is a vascular adhesion molecule induced by several mediators including pro-inflammatory cytokines like TNFα, reactive oxygen species (ROS), turbulent blood flow, microbial stimulation of toll-like receptors, high glucose concentrations, and shear stress [19–23]. Activation for binding of leukocytes with VCAM1 relies on local microenvironmental factors including chemokine profile and has been shown to play a role in endothelial integrity and function in pathological states, such as atherosclerosis [24–30]. Similar to cardiovascular events, studies have investigated the role of VCAM1 in cerebrovascular events including ischemic stroke. In this study, we take a novel approach by sampling both intracranial and systemic VCAM1 from human patients during ELVO stroke to investigate how it relates to comorbid conditions, stroke severity, functional recovery, and protein-protein signaling pathways.

Soluble VCAM1 has been reported to be a biomarker of endothelial dysfunction associated with both hypertension and atherosclerosis [27, 31–34]. Increased levels of soluble VCAM1 have been observed in patients with primary hypertension and VCAM1 levels have been shown to be reduced after antihypertensive therapy [35–38]. Tchalla et al. studied levels of circulating soluble VCAM1 in an adult population and reported elevated levels may serve as a marker of blood flow dysregulation due to hypertension-induced endothelial damage [31]. Within our cohort, those with HTN had significantly higher levels of both intracranial and systemic VCAM1 when compared to those without HTN, further supporting this relationship. Interestingly, our data demonstrate that the relationship between HTN and VCAM1 is not only limited to systemic arterial circulation but is also found locally at the site of infarct. VCAM1 is a key player in facilitating atherosclerosis by aiding in the local attachment and transmigration of inflammatory cells through the vascular endothelium [39–42]. Interestingly, we found no significant relationship between systemic or intracranial VCAM1 levels and the presence of hypercholesterolemia. Other comorbid conditions have also been reported; for example, in a population with coronary artery disease, levels of soluble VCAM1 were significantly higher in those who smoked as compared to those who did not [43] though in our ELVO cohort we found no significant relationship with VCAM1 levels and smoking status.

The Chongqing Stroke Study reported that ischemic stroke patients with neurological deterioration had higher levels of soluble ICAM1 but not soluble VCAM1 compared to patients without neurological deterioration based on the NIHSS [44]. Another study reported increased levels of VCAM1 in patients with thromboembolic stroke compared to controls [1]. In that study, there was no significant correlation between levels of soluble VCAM1 and stroke severity or disability based on NIHSS conducted at time of blood sampling (within 24 h of stroke symptom onset). Blum et al. investigated the association between NIHSS on admission and 4 days later with levels of cell adhesion molecules in acute ischemic patients [45]. They reported that in 19 out of 23 patients who improved after an acute cerebral event, there was a significant decrease in the NIHSS which was accompanied by a significant decrease in the cell adhesion molecules E-selectin, ICAM1, and VCAM1. In contrast to our results, those data demonstrate that for patients who experienced clinical improvement in the first few days after insult, the expression of cell adhesion molecules was reduced.

Our study assessed NIHSS in three ways. First, we analyzed the relationship between systemic and intracranial VCAM1 levels on NIHSS at admission and discharge separately, based on their respective median NIHSS. We found that increased levels of systemic VCAM1 was significantly associated with a higher NIHSS on discharge. Increased levels of intracranial VCAM1 were also found to be related to higher NIHSS on discharge, however, this was not significant. Additionally, increased levels of both systemic and intracranial VCAM1 were related to higher NIHSS on admittance; however, these values did not reach statistical significance (*p*=0.059 and *p*=0.058, respectively). Secondly, we looked at VCAM1 expression levels as they relate to the %∆NIHSS by linear regression analysis. We used %∆NIHSS to capture the improvement in stroke severity between admittance and discharge. We found that both systemic and intracranial VCAM1 levels were significantly higher in subjects with the smallest %∆NIHSS revealing that higher VCAM1 levels is positively correlated with poorer functional recovery after thrombectomy. Lastly, we used statistical quartiles to compare the 10 mildest stroke deficits based on NIHSS with the 10 most severe strokes injuries based on NIHSS. We found that increased levels of systemic VCAM1 were significantly higher in the severe category compared to the mild category. In comparison to Blum et al., our study differs as it captures infarct in-progress data rather than in the recovery phase.

It has also been reported that in patients with acute cerebral ischemia, plasma levels of VCAM1 can remain elevated over a 3-month period, showing a protracted inflammation in patients who have experienced cerebrovascular disease [10]. A longitudinal prospective study on soluble adhesion molecules after cerebral infarction reported that soluble VCAM1 reached its maximum plasma levels at 5 days after stroke [46]. We chose to investigate whether or not VCAM1 levels were correlated with infarct time (time from last known normal) and interestingly found no significant relationships. However, this may be due to the narrower window of time in which a thrombectomy occurs after the onset of occlusion as compared to a 3-month period used in other studies. These results demonstrate that levels of VCAM1 in our cohort are not dependent on infarct time, although comorbid inflammatory conditions may affect this timeline. Our data provide an acute snapshot of the in-progress infarct rather than the recovery phase after stroke. Taken together, these studies provide insight into the timeline of VCAM1 activation/inactivation in the acute event as well as in the post-stroke recovery time and how that directly affects stroke severity and recovery. Of note, our study investigated solely ELVOs in subjects during their stroke undergoing thrombectomy, whereas several prior studies capture data on a broader population with a variety of ischemic insults including ELVO [10, 46].

The goal of recanalization after stroke is to minimize tissue death and salvage penumbra by reducing infarct volume. Past studies demonstrated final infarct volume may be used as a surrogate marker of functional outcome and recovery [47, 48]. To our knowledge, we are the first group to demonstrate the relationship between VCAM1 and both infarct volume and edema volume as intracranial VCAM1 levels were significantly predictive of both infarct volume and edema volume. Although non-significant, increased systemic levels of VCAM1 were also related to infarct volume and edema volume. These data may reflect the consequences of endothelial dysfunction and damage as they relate to a direct measure of stroke severity and outcome. In addition, these data may assist in predicting treatment modalities such as the need for decompressive hemicraniectomies due to strokes with large infarct and edema volumes.

One of the main objectives of this study was to investigate VCAM1 in context to proteomic signaling networks. First, we wanted to compare our VCAM1 data to a well-known and well-reported inflammatory cytokine, IL-6. It has been shown that higher levels of circulating IL-6 are associated with stroke risk factors and increased risk of stroke [16)]. For example, a previous study demonstrated that hypomethylation of IL-6 gene increases the risk of essential hypertension [49]. Our study found systemic VCAM1 was significantly related to both systemic and intracranial IL-6 and that intracranial VCAM1 was significantly related to intracranial IL-6. Our study also reports significant relationships between VCAM1 and HTN, which may corroborate the interplay between IL-6 and VCAM1 with HTN. Additionally, peak plasma levels of IL-6 in the first week of ischemic stroke significantly related to infarct volume at 5-7 days post-stroke, as well as clinical outcome at 3 months [17]. Our data supports existing literature and adds to it by providing data on intracranial levels and potential intracranial relationships of IL-6 with VCAM1.

To further address the objective of uncovering signaling networks, robust regression and STRING analyses were utilized. Robust regressions identified systemic and intracranial proteins related to VCAM1 that were most predictive of infarct volume and edema volume. Once identified, these proteins were used as the input for STRING analysis. The STRING database allows investigators to explore physical and functional associations between proteins by utilizing genome-wide proteomic connectivity integrations focused on a specific biological process of interest [50]. Publications based on STRING demonstrate the utility for synthesizing text mining and experimental evidence to provide probabilities of viral and proteomic interactions [51]. These analyses uncover functions of relevant network pathways and provide information on biological predictors which are of particular interest to our research goal. From STRING, we captured the top 10 biologic functions for each network analyzed shown in Table 3. Common biological functions included “immune response,” “defense response,” “cytokine-mediated signaling pathway,” and “regulation of T cell activation.” These functions provide a useful snapshot of the biologic processes underway in both systemic and intracranial arterial blood at the time of mechanical thrombectomy.

For each VCAM1 network, some proteins were associated with all of the top 10 biological functions. For example, for the intracranial VCAM1 and infarct volume network, C-C motif chemokine ligand 2 (CCL2) was associated with each of the top 10 biological functions of that network. Prior literature demonstrated that genetic predisposition to higher levels of CCL2 is associated with higher risk of ischemic stroke [52]. Further, a recent study also found elevated CCL2 expression in a rat model of middle cerebral artery occlusion, and when CCL2 inhibitors were administered, there was a significant reduction in the inflammatory response and neurological impairment [53]. Our findings support these studies by reporting intracranial data along with networks of associated proteins that are related to objective clinical measures such as infarct volume.

Intracranial and systemic sampling of arterial blood at the time of thrombectomy provides an interesting physiologic/pathologic snapshot of ischemic stroke. Moreover, understanding the local microenvironment at the lesion site may allow therapeutics to be developed for intracranial administration during the thrombectomy. As increased VCAM1 levels were predictive of infarct volume and edema volume, future studies will aim to reverse translate these results into experimental stroke models with intention of neutralizing VCAM1 in the acute phase of ischemic stroke. Our study offers many starting points for analyses similar to Li et al. which utilize reverse translation for development of therapeutics [53]. In this study, we also used a model to identify protein-protein networks that were predictive of infarct volume and infarct edema. These data provide insight into the larger picture of signaling pathways and physiologic functions that may predict stroke severity and further our understanding of stroke diagnostics and prognostics. In addition, these data provide practical therapeutic targets for drug development and drug repurposing and will be the focus of future translational studies.

One limitation of our study is the sample size. After exclusion, we studied proteomic data from 40 stroke subjects. Thus, this is a preliminary study that will be validated with increasing enrollment into our tissue bank and registry. Although we found significance in many of our analyses, there are potential confounding variables as well as inherent heterogeneity which may act to limit conclusions. These include the spectrum of stroke severity, ischemic injury timeline, and comorbid conditions. In addition, we must also be aware of potential variations in basal level of protein expression and proteases for each subject. Another potential limitation to our cohort is the larger proportion of female as compared to male subjects. Sex differences are well known in stroke and an expanding study population will help address this discrepancy in our patient demographics [54]. Lastly, this study is limited by its geographic location. Our hospital in Central Kentucky serves a population primarily Caucasian with comorbidities common to the region. Many of these limitations highlight the utility of multi-institutional/multi-national collaborations. As our thrombectomy registry grows and collaborations develop, we will be able to combat the variances and limitations in these data as well as conduct subset statistical analyses with larger power and generalizability.

With data on 40 subjects, one potential critique is that this study suffered from low power when investigating differences in VCAM1 expression by comorbidities. Assuming the comorbidities were evenly distributed throughout the sample, this study had 80% power to detect a Cohen’s *d* effect size of 0.90 using a two tailed *t* test where *α* = .05. According to conventional cutoffs, this represents a large effect. Thus, it should not be assumed that comorbidities with non-significant VCAM1 differences reported here are not different in the population—this study was not powered to find differences in the small to medium range. Future studies utilizing larger samples will be helpful in determining how VCAM1 expression differs according to the presence of comorbidities.

## Conclusion

The current study provides novel data on systemic and intracranial VCAM1 in relation to stroke comorbidities, stroke severity, and recovery, as well as the role VCAM1 plays in complex protein-protein signaling pathways. These data will allow future studies to reverse translate into animal models for drug development. This study also serves as an example of how contemporary analytical tools from human data can be utilized to better predict stroke as well as uncover novel targets for much needed therapeutics.

## Supplementary Information

**Additional file 1.** Supplemental Table 1: List of all proteins by abbreviation and full name along with synopsis of each protein’s function taken directly from STRING.

## Data Availability

Data are available upon request. Please contact the corresponding author (KRP) for details.

## Abbreviations

ELVO: Emergent large vessel occlusion
VCAM1: Vascular cell adhesion molecule 1
BACTRAC: Blood and clot thrombectomy registry and collaboration
NIHSS: National Institute of Health Stroke Scale
IRB: Institutional Review Board
PMN: Polymorphonuclear cells
MT: Mechanical thrombectomy
BMI: Body mass index
LKN: Last known normal
ABG: Arterial blood gas
HTN: Hypertension
SD: Standard deviation
FDR: False discovery rate
ROS: Reactive oxygen species
TICI: Thrombolysis in cerebral infarction
CTA: Computed tomography angiogram
IL6: Interleukin 6
CCL2: C-C motif chemokine ligand 2
TNF: Tumor necrosis factor
