# Supplementary material for: Intracranial VCAM1 at time of mechanical thrombectomy predicts ischemic stroke severity
Source: J Neuroinflammation. 2021 May 11;18:109. doi: 10.1186/s12974-021-02157-4 (PMC8111916; doi:10.1186/s12974-021-02157-4)
Supplement: Supplementary file 1 — Additional file 1. Supplemental Table 1: List of all proteins by abbreviation and full name along with synopsis of each protein’s function taken directly from STRING. [file 12974_2021_2157_MOESM1_ESM.docx]

**Supplemental Table 1**: List of all proteins by abbreviation and full name along with synopsis of each protein’s function taken directly from STRING.

| Protein | Full Name and Description |
| --- | --- |
| VCAM1 | Vascular cell adhesion protein 1; Important in cell-cell recognition. Appears to function in leukocyte-endothelial cell adhesion. Interacts with integrin alpha-4/beta-1 (ITGA4/ITGB1) on leukocytes, and mediates both adhesion and signal transduction. The VCAM1/ITGA4/ITGB1 interaction may play a pathophysiologic role both in immune responses and in leukocyte emigration to sites of inflammation; C2-set domain containing (739 aa) |
| MMP10 | Stromelysin-2; Can degrade fibronectin, gelatins of type I, III, IV, and V; weakly collagens III, IV, and V. Activates procollagenase; Belongs to the peptidase M10A family (476 aa) |
| IL18 | Interleukin-18; Augments natural killer cell activity in spleen cells and stimulates interferon gamma production in T-helper type I cells; Belongs to the IL-1 family (193 aa) |
| IL10RB | Interleukin-10 receptor subunit beta; Shared cell surface receptor required for the activation of five class 2 cytokines: IL10, IL22, IL26, IL28, and IFNL1. The IFNLR1/IL10RB dimer is a receptor for the cytokine ligands IFNL2 and IFNL3 and mediates their antiviral activity. The ligand/receptor complex stimulate the activation of the JAK/STAT signaling pathway leading to the expression of IFN-stimulated genes (ISG), which contribute to the antiviral state (325 aa) |
| APOM | Apolipoprotein M; Probably involved in lipid transport. Can bind sphingosine-1-phosphate, myristic acid, palmitic acid and stearic acid, retinol, all-trans-retinoic acid and 9-cis-retinoic acid; Apolipoproteins (188 aa) |
| VASN | Vasorin; May act as an inhibitor of TGF-beta signaling (673 aa) |
| IL20RA | Interleukin-20 receptor subunit alpha; The IL20RA/IL20RB dimer is a receptor for IL19, IL20 and IL24. The IL20RA/IL10RB dimer is a receptor for IL26; Interleukin receptors (553 aa) |
| DPP4 | Dipeptidyl peptidase 4; Cell surface glycoprotein receptor involved in the costimulatory signal essential for T-cell receptor (TCR)-mediated T-cell activation. Acts as a positive regulator of T-cell coactivation, by binding at least ADA, CAV1, IGF2R, and PTPRC. Its binding to CAV1 and CARD11 induces T-cell proliferation and NF- kappa-B activation in a T-cell receptor/CD3-dependent manner. Its interaction with ADA also regulates lymphocyte-epithelial cell adhesion. In association with FAP is involved in the pericellular proteolysis of the extracellular matrix (ECM), the migration and in [...] (766 aa) |
| CST3 | Cystatin-C; As an inhibitor of cysteine proteinases, this protein is thought to serve an important physiological role as a local regulator of this enzyme activity; Belongs to the cystatin family (146 aa) |
| AOC3 | Membrane primary amine oxidase; Cell adhesion protein that participates in lymphocyte extravasation and recirculation by mediating the binding of lymphocytes to peripheral lymph node vascular endothelial cells in an L-selectin-independent fashion. Has semicarbazide-sensitive (SSAO) monoamine oxidase activity. May play a role in adipogenesis (763 aa) |
| CXCL9 | C-X-C motif chemokine 9; Cytokine that affects the growth, movement, or activation state of cells that participate in immune and inflammatory response. Chemotactic for activated T-cells. Binds to CXCR3; Belongs to the intercrine alpha (chemokine CxC) family (125 aa) |
| CD59 | CD59 glycoprotein; Potent inhibitor of the complement membrane attack complex (MAC) action. Acts by binding to the C8 and/or C9 complements of the assembling MAC, thereby preventing incorporation of the multiple copies of C9 required for complete formation of the osmolytic pore. This inhibitor appears to be species-specific. Involved in signal transduction for T-cell activation complexed to a protein tyrosine kinase; Blood group antigens (128 aa) |
| CCL2 | C-C motif chemokine 2; Chemotactic factor that attracts monocytes and basophils but not neutrophils or eosinophils. Augments monocyte anti-tumor activity. Has been implicated in the pathogenesis of diseases characterized by monocytic infiltrates, like psoriasis, rheumatoid arthritis or atherosclerosis. May be involved in the recruitment of monocytes into the arterial wall during the disease process of atherosclerosis; Belongs to the intercrine beta (chemokine CC) family (99 aa) |
| LILRB2 | Leukocyte immunoglobulin-like receptor subfamily B member 2; Receptor for class I MHC antigens. Recognizes a broad spectrum of HLA-A, HLA-B, HLA-C and HLA-G alleles. Involved in the down-regulation of the immune response and the development of tolerance. Competes with CD8A for binding to class I MHC antigens. Inhibits FCGR1A-mediated phosphorylation of cellular proteins and mobilization of intracellular calcium ions (598 aa) |
| IGFBP6 | Insulin-like growth factor-binding protein 6; IGF-binding proteins prolong the half-life of the IGFs and have been shown to either inhibit or stimulate the growth promoting effects of the IGFs on cell culture. They alter the interaction of IGFs with their cell surface receptors (240 aa) |
| ITGAM | Integrin alpha-M; Integrin ITGAM/ITGB2 is implicated in various adhesive interactions of monocytes, macrophages and granulocytes as well as in mediating the uptake of complement-coated particles. It is identical with CR-3, the receptor for the iC3b fragment of the third complement component. It probably recognizes the R-G-D peptide in C3b. Integrin ITGAM/ITGB2 is also a receptor for fibrinogen, factor X and ICAM1. It recognizes P1 and P2 peptides of fibrinogen gamma chain. Regulates neutrophil migration. In association with beta subunit ITGB2/CD18, required for CD177-PRTN3-mediated act [...] (1153 aa) |
| NCAM1 | Neural cell adhesion molecule 1; This protein is a cell adhesion molecule involved in neuron-neuron adhesion, neurite fasciculation, outgrowth of neurites, etc; CD molecules (884 aa) |
| MEGF9 | Multiple epidermal growth factor-like domains protein 9; Multiple EGF like domains 9 (602 aa) |
| SLAMF1 | Signaling lymphocytic activation molecule; Self-ligand receptor of the signaling lymphocytic activation molecule (SLAM) family. SLAM receptors triggered by homo- or heterotypic cell-cell interactions are modulating the activation and differentiation of a wide variety of immune cells and thus are involved in the regulation and interconnection of both innate and adaptive immune response. Activities are controlled by presence or absence of small cytoplasmic adapter proteins, SH2D1A/SAP and/or SH2D1B/EAT-2. SLAMF1-induced signal- transduction events in T-lymphocytes are different from thos [...] (335 aa) |
| CCL18 | C-C motif chemokine 18; Chemotactic factor that attracts lymphocytes but not monocytes or granulocytes. May be involved in B-cell migration into B-cell follicles in lymph nodes. Attracts naive T-lymphocytes toward dendritic cells and activated macrophages in lymph nodes, has chemotactic activity for naive T-cells, CD4+ and CD8+ T-cells and thus may play a role in both humoral and cell-mediated immunity responses; Chemokine ligands (89 aa) |
| ANG | Angiogenin; Binds to actin on the surface of endothelial cells; once bound, angiogenin is endocytosed and translocated to the nucleus. Stimulates ribosomal RNA synthesis including that containing the initiation site sequences of 45S rRNA. Cleaves tRNA within anticodon loops to produce tRNA-derived stress-induced fragments (tiRNAs) which inhibit protein synthesis and triggers the assembly of stress granules (SGs). Angiogenin induces vascularization of normal and malignant tissues. Angiogenic activity is regulated by interaction with RNH1 in vivo; Ribonuclease A family (147 aa) |
| CHL1 | Neural cell adhesion molecule L1-like protein; Extracellular matrix and cell adhesion protein that plays a role in nervous system development and in synaptic plasticity. Both soluble and membranous forms promote neurite outgrowth of cerebellar and hippocampal neurons and suppress neuronal cell death. Plays a role in neuronal positioning of pyramidal neurons and in regulation of both the number of interneurons and the efficacy of GABAergic synapses. May play a role in regulating cell migration in nerve regeneration and cortical development. Potentiates integrin-dependent cell migration [...] (1224 aa) |
| ITGAM | Integrin alpha-M; Integrin ITGAM/ITGB2 is implicated in various adhesive interactions of monocytes, macrophages and granulocytes as well as in mediating the uptake of complement-coated particles. It is identical with CR-3, the receptor for the iC3b fragment of the third complement component. It probably recognizes the R-G-D peptide in C3b. Integrin ITGAM/ITGB2 is also a receptor for fibrinogen, factor X and ICAM1. It recognizes P1 and P2 peptides of fibrinogen gamma chain. Regulates neutrophil migration. In association with beta subunit ITGB2/CD18, required for CD177-PRTN3-mediated act [...] (1153 aa) |
| SORT1 | Sortilin; Functions as a sorting receptor in the Golgi compartment and as a clearance receptor on the cell surface. Required for protein transport from the Golgi apparatus to the lysosomes by a pathway that is independent of the mannose-6-phosphate receptor (M6PR). Also required for protein transport from the Golgi apparatus to the endosomes. Promotes neuronal apoptosis by mediating endocytosis of the proapoptotic precursor forms of BDNF (proBDNF) and NGFB (proNGFB). Also acts as a receptor for neurotensin. May promote mineralization of the extracellular matrix during osteogenic differ [...] (831 aa) |
| APOM | Apolipoprotein M; Probably involved in lipid transport. Can bind sphingosine-1-phosphate, myristic acid, palmitic acid and stearic acid, retinol, all-trans-retinoic acid and 9-cis-retinoic acid; Apolipoproteins (188 aa) |
| MMP10 | Stromelysin-2; Can degrade fibronectin, gelatins of type I, III, IV, and V; weakly collagens III, IV, and V. Activates procollagenase; Belongs to the peptidase M10A family (476 aa) |
| SLAMF1 | Signaling lymphocytic activation molecule; Self-ligand receptor of the signaling lymphocytic activation molecule (SLAM) family. SLAM receptors triggered by homo- or heterotypic cell-cell interactions are modulating the activation and differentiation of a wide variety of immune cells and thus are involved in the regulation and interconnection of both innate and adaptive immune response. Activities are controlled by presence or absence of small cytoplasmic adapter proteins, SH2D1A/SAP and/or SH2D1B/EAT-2. SLAMF1-induced signal- transduction events in T-lymphocytes are different from thos [...] (335 aa) |
| CD274 | Programmed cell death 1 ligand 1; Involved in the costimulatory signal, essential for T- cell proliferation and production of IL10 and IFNG, in an IL2- dependent and a PDCD1-independent manner. Interaction with PDCD1 inhibits T-cell proliferation and cytokine production; Belongs to the immunoglobulin superfamily. BTN/MOG family (290 aa) |
| CCL25 | C-C motif chemokine 25; Potentially involved in T-cell development. Recombinant protein shows chemotactic activity on thymocytes, macrophages, THP-1 cells, and dendritics cells but is inactive on peripheral blood lymphocytes and neutrophils. Binds to CCR9. Isoform 2 is an antagonist of isoform 1. Binds to atypical chemokine receptor ACKR4 and mediates the recruitment of beta-arrestin (ARRB1/2) to ACKR4; Belongs to the intercrine beta (chemokine CC) family (150 aa) |
| CCL5 | C-C motif chemokine 5; Chemoattractant for blood monocytes, memory T-helper cells and eosinophils. Causes the release of histamine from basophils and activates eosinophils. May activate several chemokine receptors including CCR1, CCR3, CCR4 and CCR5. One of the major HIV-suppressive factors produced by CD8+ T-cells. Recombinant RANTES protein induces a dose-dependent inhibition of different strains of HIV-1, HIV-2, and simian immunodeficiency virus (SIV). The processed form RANTES(3-68) acts as a natural chemotaxis inhibitor and is a more potent inhibitor of HIV-1- infection. The secon [...] (91 aa) |
| SLAMF1 | Signaling lymphocytic activation molecule; Self-ligand receptor of the signaling lymphocytic activation molecule (SLAM) family. SLAM receptors triggered by homo- or heterotypic cell-cell interactions are modulating the activation and differentiation of a wide variety of immune cells and thus are involved in the regulation and interconnection of both innate and adaptive immune response. Activities are controlled by presence or absence of small cytoplasmic adapter proteins, SH2D1A/SAP and/or SH2D1B/EAT-2. SLAMF1-induced signal- transduction events in T-lymphocytes are different from thos [...] (335 aa) |
| MBL2 | Mannose-binding protein C; Calcium-dependent lectin involved in innate immune defense. Binds mannose, fucose and N-acetylglucosamine on different microorganisms and activates the lectin complement pathway. Binds to late apoptotic cells, as well as to apoptotic blebs and to necrotic cells, but not to early apoptotic cells, facilitating their uptake by macrophages. May bind DNA; Collectins (248 aa) |
| F7 | Coagulation factor VII; Initiates the extrinsic pathway of blood coagulation. Serine protease that circulates in the blood in a zymogen form. Factor VII is converted to factor VIIa by factor Xa, factor XIIa, factor IXa, or thrombin by minor proteolysis. In the presence of tissue factor and calcium ions, factor VIIa then converts factor X to factor Xa by limited proteolysis. Factor VIIa will also convert factor IX to factor IXa in the presence of tissue factor and calcium; Gla domain containing (466 aa) |
| IL15RA | Interleukin 15 receptor subunit alpha; CD molecules (353 aa) |
| CD274 | Programmed cell death 1 ligand 1; Involved in the costimulatory signal, essential for T- cell proliferation and production of IL10 and IFNG, in an IL2- dependent and a PDCD1-independent manner. Interaction with PDCD1 inhibits T-cell proliferation and cytokine production; Belongs to the immunoglobulin superfamily. BTN/MOG family (290 aa) |
| CCL18 | C-C motif chemokine 18; Chemotactic factor that attracts lymphocytes but not monocytes or granulocytes. May be involved in B-cell migration into B-cell follicles in lymph nodes. Attracts naive T-lymphocytes toward dendritic cells and activated macrophages in lymph nodes, has chemotactic activity for naive T-cells, CD4+ and CD8+ T-cells and thus may play a role in both humoral and cell-mediated immunity responses; Chemokine ligands (89 aa) |
| CCL11 | Eotaxin; In response to the presence of allergens, this protein directly promotes the accumulation of eosinophils, a prominent feature of allergic inflammatory reactions. Binds to CCR3; Chemokine ligands (97 aa) |
| FAP | Prolyl endopeptidase FAP; Cell surface glycoprotein serine protease that participates in extracellular matrix degradation and involved in many cellular processes including tissue remodeling, fibrosis, wound healing, inflammation and tumor growth. Both plasma membrane and soluble forms exhibit post-proline cleaving endopeptidase activity, with a marked preference for Ala/Ser-Gly-Pro-Ser/Asn/Ala consensus sequences, on substrate such as alpha-2-antiplasmin SERPINF2 and SPRY2. Degrade also gelatin, heat-denatured type I collagen, but not native collagen type I and IV, vibronectin (760 aa) |
| TSLP | Thymic stromal lymphopoietin; Isoform 1: Cytokine that induces the release of T-cell- attracting chemokines from monocytes and, in particular, enhances the maturation of CD11c(+) dendritic cells. Can induce allergic inflammation by directly activating mast cells (159 aa) |
| CSF1 | Macrophage colony-stimulating factor 1; Cytokine that plays an essential role in the regulation of survival, proliferation and differentiation of hematopoietic precursor cells, especially mononuclear phagocytes, such as macrophages and monocytes. Promotes the release of proinflammatory chemokines, and thereby plays an important role in innate immunity and in inflammatory processes. Plays an important role in the regulation of osteoclast proliferation and differentiation, the regulation of bone resorption, and is required for normal bone development (554aa) |
